# Miscellany

**Published:** 1891-01

**Authors:** 


### Miscellany.

THE MONIST (philosophical quarterly) for January, 1891, contains: The Architecture of Theories, by Charles S. Pierce. Illustrative Studies in Criminal Anthropology. (1). "La Bête Humaine" and Criminal Anthropology. (2). Psychiatry and Criminal Anthropology. *a.* Secretion of Criminals. *b.* Power of Smell. *c.* Taste. *d.* Walk. *e.* Gestures. *f.* Morphological Anomalies, Skeleton, Skull, Wrinkles. *g.* Tattooing, by Prof. Cesare Lombroso. The Squaring of the Circle. The history of the problem from the most ancient times to the present day, by Hermann Schubert. The Criterion of Truth. A Dissertation on the Method of Verifications, by Dr. Paul Carus. Five Souls with but a Single Thought—The Psychology of the Star-Fish, by Carus Sterne. German Philosophy in the Nineteenth Century, by Prof. Friedrich Jodl. Recent French Philosophical Works, by Lucien Arréat. Book Reviews. Contents of the Philosophical Periodicals of America and Europe. (The Open Court Pub. Co., Chicago.)

---

An Interesting Literary Personality.—The January number of the *Cosmopolitan* contains the first of two parts of the new novel by Mrs. Van Rensselaer Cruger, whose "Diplomat's Diary" and "A Successful Man," the latter first published in the *Cosmopolitan*, excited so much comment both in this country and in Europe. She is undoubtedly the most interesting personality who has appeared in the literary field since the entrée of Amelie Rives; but, unlike Miss Rives, who was brought up amidst the country surroundings of a Virginia home, and who was a girl in her teens when she began to write, Mrs. Cruger has been for years a leader of New York society, and has spent a couple of Winters at some of the most famous courts of Europe, and, while yet a very young woman, has enjoyed the richest experiences of life.

With a view to the introduction of the *Cosmopolitan* to the readers of this journal, we propose to do even better than the very low price of the magazine. To those who have never been subscribers to the *Cosmopolitan*, we will furnish the Buffalo Medical and Surgical Journal and that magazine for $3 for the two.

---

Famous yet Unknown.—*Wives who are hidden by their husband's fame, yet who are wonderful women.*—The wife of a famous man will oftimes be completely hidden by the dazzle of her husband's fame, and it is astonishing how little is known of those women whose husband's names are household words throughout the country. While the newspapers teem with the name of Thomas A. Edison, nothing is comparatively known or heard of Mrs. Edison. Every newspaper reader knows the name of Chauncey M. Depew, but of Mrs. Depew only the most casual reference is made. Even in England, no one ever hears of Lady Tennyson, or of Mrs. Gladstone. And the same is true of the wives of such men as P. T. Barnum, Will Carleton, John Wanamaker, Spurgeon, W. D. Howells, Dr. Talmage, "Mark Twain," and James G. Blaine. Often these very wives have been the makers of their husband's careers. Their portraits are even less known than their lives. In a splendid series to be called "Unknown Wives of Well-known Men," *The Ladies' Home Journal*, of Philadelphia, will, during next year, sketch all these women and others, presenting their portraits, in many cases, for the first time to the public.

A regular physician wishes to form a partnership with some prominent physician in Buffalo. He is thirty-seven years old, and has been sixteen years in practice. Has been a private student with the most able gynecologists and abdominal surgeons in England and on the Continent. Wishes to confine himself principally to diseases of women and abdominal surgery. Will correspond or visit and give the best references. Address C., Buffalo Medical and Surgical Journal.

---

The Literary Digest is an almost indispensable weekly visitor to a gentleman's library table. It contains political and sociological reviews and selections; also extracts from all the leading newspapers relating to education, literature and art, as well as science, religion, and miscellaneous departments. The department allotted to The Press is especially to be commended as affording to a busy man an opportunity of knowiug the most important views of the best domestic and foreign journals on current topics; and, finally, it gives an index of periodical literature that is of great value. It is published by Funk & Wagnalls, 18 and 20 Astor place, New York.

---

Nearly Ready.— Transactions of the American Association of Obstetricians and Gynecologists. Volume III., for 1890. A handsome book of nearly 500 octavo pages, profusely illustrated, and containing the latest literature on the most important questions relating to abdominal surgery, obstetrics, and gynecology. Price, cloth, $5.00 ; half Russia, $6.00. Orders should be placed promptly, as the edition is limited. Address the printer, William J. Dornan, 100 North Seventh street, Philadelphia, Pa., or William Warren Potter, M. D., Secretary, 284 Franklin street, Buffalo, N. Y.

---

The Open Court Publishing Co., of Chicago, will publish immediately, in two handsomely bound and printed volumes, a new authorized translation of Gustav Freytag's well-known novel, "The Lost Manuscript." This is regarded by critics as the most charming of the famous German writer's works.

